# Quitline Use and Outcomes among Callers with and without Mental Health Conditions: A 7-Month Follow-Up Evaluation in Three States

**DOI:** 10.1155/2015/817298

**Published:** 2015-07-26

**Authors:** Katrina A. Vickerman, Gillian L. Schauer, Ann M. Malarcher, Lei Zhang, Paul Mowery, Chelsea M. Nash

**Affiliations:** ^1^Alere Wellbeing, Inc., 999 Third Avenue, Suite 2000, Seattle, WA 98104, USA; ^2^Carter Consulting, Inc., Contractor to Office on Smoking and Health, Centers for Disease Control and Prevention, 4770 Buford Highway NE, MS F-79, Atlanta, GA 30341, USA; ^3^Office on Smoking and Health, Centers for Disease Control and Prevention, 4770 Buford Highway, NE MS F-79, Atlanta, GA 30341, USA; ^4^Biostatistics, Inc., 1738 Illehaw Drive, Sarasota, FL 34239, USA

## Abstract

*Objectives*. To examine abstinence outcomes among tobacco users with and without a reported mental health condition (MHC) who enrolled in state tobacco quitline programs. *Methods*. Data were analyzed from a 7-month follow-up survey (response rate: 41% [3,132/7,459]) of three state-funded telephone quitline programs in the United States that assessed seven self-reported MHCs at quitline registration. We examined 30-day point prevalence tobacco quit rates for callers with any MHC versus none. Data were weighted to adjust for response bias and oversampling. Multivariable logistic regression was used to examine cessation outcomes. *Results*. Overall, 45.8% of respondents reported ≥1 MHC; 57.4% of those reporting a MHC reported ≥2 MHCs. The unadjusted quit rate for callers with any MHC was lower than for callers with no MHC (22.0% versus 31.0%, *P* < 0.001). After adjusting for demographics, nicotine dependence, and program engagement, callers reporting ≥1 MHC were less likely to be abstinent at follow-up (adjusted OR = 0.63, 95% CI = 0.51–0.78, *P* < 0.001). *Conclusions*. More intensive or tailored quitline programs may need to be developed among callers with MHCs as their quit rates appear to be lower than callers without MHCs.

## 1. Introduction

Individuals with mental health conditions (MHCs) are twice as likely to smoke compared to the general population [1] with smoking prevalence varying from 34% to over 60% depending on specific diagnosis [2, 3]. Individuals with MHCs are also more likely to die prematurely—up to 25 years earlier on average in some populations [4]—and, similar to the general population, tobacco-related conditions such as heart disease and cancer are the leading causes of death among individuals with MHCs [4].

Smoking rates have significantly declined over the past decade among the general population [5], but rates have not declined among individuals with MHCs [6]. Although studies have shown that evidence-based treatment increases cessation among persons with MHCs [7–10], individuals with MHCs may have unique treatment needs [2, 11] and, based on epidemiological evidence, have a lower rate of successful cessation than those without MHCs [1, 12]. There is growing evidence that, on average, quitting tobacco does not negatively impact long-term psychological functioning for persons with MHCs—a concern often raised by providers [13]. In fact, treatment may reduce MHC symptoms and improve functioning [14–17].

State tobacco quitlines in the United States provide evidence-based tobacco cessation treatment to more than 400,000 smokers annually, including free phone-based cessation counseling and often access to nicotine replacement therapy (NRT) [18, 19]. Approximately one-fourth of quitline callers meet criteria for major Depression [20] and approximately half or greater may have a MHC [21, 22]. Compared to those who try to quit with no support, quitline counseling increases the odds of quitting by 60% in the general population [11] and is a cost-effective public health intervention [23–27]. Quitlines may be a particularly valuable resource for individuals with MHCs because they reduce barriers to care (i.e., cost and transportation) [28]. Little is known about the effectiveness of quitline counseling for individuals with MHCs. Lower quit rates for callers with a MHC have been reported in several conference presentations [21, 22, 29], whereas other researchers found no differences in quit rates for MHC and non-MHC populations [30] or for callers with and without a positive Depression screen [27]. In the two published studies examining MHCs among quitline callers, callers to the California Smokers' Helpline with major Depression were less likely to quit at two-month follow-up (19% versus 28%) [20], and callers to the New York State Smokers' Quitline who were heavy drinkers had lower abstinence rates at two-week follow-up compared to moderate drinkers [31]. Quit rates among quitline callers with other MHCs, such as Attention Deficit Hyperactivity Disorder, Bipolar Disorder, Generalized Anxiety Disorder, Posttraumatic Stress Disorder, and Schizophrenia, have not been published.

Accordingly, the current study examines characteristics and quit outcomes for callers to three state quitlines who reported on seven MHCs at program registration. We hypothesized that callers with MHCs would also have other characteristics that may make it more challenging for them to quit, such as higher tobacco dependence and lower socioeconomic status [1, 11, 32] and that callers with MHCs would have lower 30-day point prevalence quit rates at 7-month follow-up after adjusting for these factors.

## 2. Methods

### 2.1. Sample Selection

This observational study includes data from four evaluation samples of quitline registrants from January 2012 through May 2013: one in Maryland, one in Nebraska, and two in North Carolina (one with January–June 2012 registrants and the other with August 2012–May 2013 registrants). These evaluations were conducted as part of each state's ongoing program outcome evaluation procedures. The states agreed to contribute their evaluation data and participate in this secondary data analysis. These state quitlines were selected because they asked a custom registration question to assess self-reported MHC status, offered a multiple-call phone-based cessation program, and had quitline services delivered and evaluations conducted by the same quitline vendor (Alere Wellbeing). After enrolling in the quitline program and completing their first coaching call, callers were selected for evaluation follow-up based on the following criteria. All tobacco users who had enrolled in the phone-based state quitline program in Maryland and Nebraska were selected for evaluation (census sample), until the final three months of the Maryland evaluation, when random sampling was used due to increased quitline call volumes, and thus an influx of individuals eligible for the evaluation. Callers who were pregnant, Medicaid-insured, or reported having a MHC were oversampled using a probability sampling scheme in the North Carolina evaluations. Additional details regarding sampling methods are presented in Table 1. For all evaluations, quitline participants were eligible for inclusion if they were English or Spanish speaking, 18 years or older, and a tobacco user at enrollment, provided a valid phone number, consented to evaluation follow-up during registration, and had completed at least one coaching call. Data weighting procedures were used to accommodate population oversampling during analysis.

### 2.2. The Quitline Program

All three states offered a multiple-call phone-based cessation program for the duration of the study time frame, which included an initial assessment and planning call plus three to four additional coaching calls, a printed quit guide, and access to the Web Coach website, an interactive web-based cessation resource designed to complement the phone program. The quitline program is based on social cognitive theory and the United States Public Health Service Clinical Practice Guidelines [11, 33]. Calls focus on creating a quit plan, using problem solving and skills development to address cravings and triggers, leveraging social support, and using cessation medications to achieve abstinence and avoid relapse. All tobacco users who were ready to quit in the next 30 days at the time of registration were eligible for the multiple-call program. A one-call program was available for callers not ready to quit or not interested in the multiple-call program. Only a small number of callers selected the one-call program, and only 98 completed a follow-up survey (2.9% of all completed surveys) during the study time frame. Accordingly, we have excluded those opting for the one-call program from these analyses. Free NRT was provided through the quitline to eligible callers in Maryland and North Carolina. Table 1 presents additional details regarding quitline services and NRT offerings during the study time frame.

### 2.3. Follow-Up Survey Administration

Evaluations were conducted in accordance with recommendations from the North American Quitline Consortium for assessing quit outcomes for state quitlines in North America [34]. Sampled participants were contacted approximately 7 months after completing their first coaching call. Participants with a valid email address who consented to being contacted via email were emailed an invitation to complete the follow-up survey online. Those who did not complete the survey after three reminder emails were then contacted by trained interviewers to complete a phone-based survey. Interviewers made at least one attempt per day to reach each participant by phone; attempts were made on up to 11 different days over approximately a 4-week period.

Across the three states, 7,646 tobacco users who enrolled in the multiple-call programs within January 2012–May 2013 were selected for evaluation; 3,262 completed the 7-month survey (response rate: 42.7%). The final sample includes 3,132 participants (40.9%) who responded to the question assessing MHC status during quitline registration and also provided their quit status at 7-month follow-up.

### 2.4. Measures

#### 2.4.1. Baseline Program Registration Data

To assess MHC status at program registration, callers were asked a behavioral health question similar to one developed by a NAQC advisory forum [35]: “Do you currently have any mental health conditions, such as Attention Deficit Hyperactivity Disorder (ADHD), Bipolar Disorder, Depression, Drug or Alcohol Use Disorder (or Substance Use Disorder; SUD), Generalized Anxiety Disorder (GAD), Posttraumatic Stress Disorder (PTSD), and Schizophrenia?” Registration agents paused briefly after each condition to allow participants to respond. We examined outcomes for callers who reported any MHC versus no MHCs. In addition, because the majority of callers with a MHC reported comorbid MHCs, we also established four mutually exclusive diagnostic groups. The groups were based on conditions that typically have the greatest impact on daily functioning and conditions that were highly comorbid in our sample, such as Depression and anxiety disorders: (Group 1) Schizophrenia or Bipolar Disorder; (Group 2) Depression, GAD, or PTSD but no report of Schizophrenia or Bipolar Disorder; (Group 3) SUD or ADHD but no report of the other conditions; and (Group 4) no MHC. Demographics (age, gender, education, race/ethnicity, and chronic health condition status [presence of asthma, diabetes, chronic obstructive pulmonary disease, and/or coronary artery disease]), baseline tobacco use data (type [cigarette, cigar, pipe, smokeless, and other], amount [cigarettes per day or CPD], time to first use after waking [TTFU]), and health insurance status (private, Medicare, Medicaid, and uninsured) were collected during quitline registration, per NAQC guidelines [36]. CPD and TTFU were used to create a nicotine dependence index. CPD was recorded on a continuous scale and categorized into four groups: (1) 0–10, (2) 11–20, (3) 21–30, and (4) 31 or more. TTFU was recorded on a 4-point scale: (1) 61 or more minutes, (2) 31–60 minutes, (3) 6–30 minutes, and (4) within 5 minutes. The index is the mean of these two 4-point scales.

#### 2.4.2. Program Engagement

The number of coaching calls completed in the program and whether NRT was sent from the quitline were recorded and examined as indicators of program engagement.

#### 2.4.3. 7-Month Follow-Up Survey

Tobacco cessation outcomes were assessed during the 7-month follow-up survey by asking respondents, “When did you last use tobacco, even a puff or a pinch? (Please do not include electronic cigarettes)”. Respondents' self-reported last tobacco use data were used to calculate 7- and 30-day point prevalence tobacco quit rates. Participants were also asked whether they had used any cessation medications to help them quit since enrolling in the quitline (NRT patch, gum, lozenge, inhaler, nasal spray, Chantix/varenicline, Zyban/bupropion/Wellbutrin, and “other” medication).

### 2.5. Analyses

Data were weighted separately to the populations eligible for the evaluation for each state for nonresponse to the 7-month survey based on age, gender, race/ethnicity, insurance status, call completion, and dependence level. Data from the three states were then combined and poststratification weights were computed to adjust for oversampling in North Carolina evaluations (by pregnancy status, presence of a MHC, and Medicaid status); all variables used during response bias weighting were also included in this step. Weights were computed using a raking macro [37]. Weighted analyses were conducted in SAS 9.3 (SAS Institute Inc., Cary, NC). The purpose of these weighting procedures was to increase the generalizability of the 7-month survey results to the entire population eligible for evaluation in these states (*N* = 28,391) by adjusting the weights of individuals' 7-month survey data to ensure that respondent characteristics were similar to population characteristics on the weighting variables listed above.

Demographics, tobacco use characteristics, program engagement, and 7-month survey outcomes were examined for callers with and without reported MHCs. Multivariable logistic regression was used to assess whether callers with any MHCs were less likely to quit for 30 days or more at follow-up compared to those without MHCs, controlling for age, gender, race/ethnicity, education, insurance status, number of calls completed, use of cessation medications reported at follow-up, and state (to account for state-level differences in tobacco control environment and quitline services). Two models were estimated; the first examined any versus no MHCs, and the second examined MHC status divided into the four mutually exclusive condition groups described above (Group 1: Schizophrenia/Bipolar Disorder; Group 2: Depression/GAD/PTSD; Group 3: SUD/ADHD; Group 4: no MHC). In the second model, each condition group was compared to no MHC and to each other.

## 3. Results

Nearly half (45.8%) of survey respondents reported one or more MHCs at baseline including: 31.9% Depression, 21.2% GAD, 13.6% Bipolar Disorder, 8.4% PTSD, 7.4% ADHD, 6.7% SUD, and 3.6% Schizophrenia. The majority (57.4%) of those reporting a MHC reported two or more comorbid MHCs (26.3% of the total sample).

Compared to those without MHCs, a higher percentage of respondents with a MHC were younger, female, White non-Hispanic, and Medicaid-insured, had less than a high school degree, had higher tobacco dependence (based on CPD and TTFU), had a chronic health condition, and completed three or more program calls (Tables 2 and 3). A lower percentage of callers with a MHC had been mailed NRT through the quitline; however, there were no differences in self-reported use of NRT since program registration (Table 3). There were no differences in type of tobacco used (Table 2) or satisfaction with the quitline (Table 3).

### 3.1. Quit Rates for Callers with and without MHCs

At the time of the 7-month survey, the unadjusted quit rate for callers with a MHC was significantly lower than for callers without a MHC (30-day point prevalence quit rates: 22.0% (95% CI = 19.5%–24.5%) versus 31.0% (95% CI = 28.4%–33.6%), *P* < 0.001) (Table 3). Multivariable logistic regression analyses confirmed that callers who reported one or more MHCs were significantly less likely to quit at follow-up (adjusted OR = 0.63, 95% CI = 0.51–0.77, *P* < 0.001) (Table 4). Callers with higher baseline tobacco dependence and Medicaid insurance (compared to private) were also less likely to quit at follow-up, and callers who completed more program calls were more likely to quit.

### 3.2. Quit Rates by MHC Group

Unadjusted 30-day point prevalence quit rates for the mutually exclusive MHC groups were as follows: Schizophrenia or Bipolar Disorder (Group 1) [19.4% (95% CI = 15.4%–23.3%)]; Depression, GAD, or PTSD without Schizophrenia or Bipolar Disorder (Group 2) [24.0% (95% CI = 20.6%–27.5%)]; SUD or ADHD without the other five conditions (Group 3) [18.3% (95% CI = 9.5%–27.1%)] (data not shown in tables). In multivariable logistic regression analyses, all three condition groups were significantly less likely to quit compared to callers with no MHC (Table 4); the three MHC groups did not significantly differ in likelihood of being quit [Group 1 versus 2: adjusted OR = 0.76 (95% CI = 0.54–1.06), *P* = 0.11; Group 1 versus 3: adjusted OR = 1.21 (95% CI = 0.58–2.51), *P* = 0.61; Group 2 versus 3: adjusted OR = 1.60 (95% CI = 0.79–3.22), *P* = 0.19].

## 4. Discussion

Among callers to three state quitline multiple-call programs, nearly half (46%) reported one of seven current MHCs, and callers reporting a MHC were significantly less likely to quit at 7-month follow-up compared to callers without a MHC. After controlling for demographics, baseline tobacco dependence, program utilization characteristics, and state, callers with a MHC had 0.6 times lower adjusted odds of being quit compared to callers without a MHC. These findings may seem to suggest that callers with MHCs are not benefiting from quitline services; however, less than 10% of people in the general population who use no support or minimal self-help successfully quit smoking [11]. Epidemiological research indicates that odds of successfully quitting may be even lower for individuals with MHCs [1, 12]. Because this was an observational study, we could not determine what the relative likelihood of quitting without assistance would have been for those with and without a MHC, so we could not determine how much the quitline intervention increased the odds of success, and whether this differed for callers with and without a MHC.

Quitline callers with MHCs also have other characteristics that have been shown in previous research to make quitting harder, including higher tobacco dependence, lower education and socioeconomic status, and having other chronic health conditions [1, 11, 32]. Given that callers with MHCs are more likely to have sociodemographic characteristics associated with higher tobacco use and greater difficulty quitting, improving treatment for callers with MHCs may also help other priority populations disproportionately impacted by tobacco use. Assessing mutually exclusive MHC groups, we found that all three condition groups differed from callers with no MHCs in multivariable models but did not differ from one another. Together with the finding that callers with any MHCs were less likely to quit than those with none, this suggests that any report of a MHC may predict increased difficulty in quitting, even though the population of individuals with any MHC is likely a heterogeneous group in terms of symptoms, stressors, and daily functioning.

Despite having characteristics that can hinder quitting, our data suggest that callers with MHCs are engaged in the quitting process. For example, callers with MHCs were more likely than those without MHCs to complete three or more program calls, which has been associated with greater quit success [24, 32, 38]. Findings indicating greater treatment engagement among those with MHCs could be due to an increased need for support and/or high motivation to change. More research is needed to assess why quitline program engagement is higher in this population.

Callers with and without MHCs were equally likely to report having used cessation medications during their quit attempt; however, fewer callers with MHCs were sent medications through the quitlines, which may have been due to NRT contraindication guidelines, eligibility criteria for the specific states, or interest in using medications not covered by the quitlines (e.g., varenicline and bupropion). It is encouraging that callers with MHCs obtained cessation medications from other sources, particularly given that callers with MHCs are more likely to have higher tobacco dependence [1, 20] and may need more intensive medication support than the general population [11, 28, 39]. Since barriers such as copays and prior authorizations can negatively impact access to cessation medication and quitting success [40–42], provision of free NRT directly through quitlines could further improve medication use for all callers. Reported medication use was not a significant predictor of quit status at 7 months in the multivariable model, which is not an unusual finding in quitline observational studies where participants self-select whether to use cessation medications [32]; this should not be interpreted as medications being unimportant in callers' quitting process.

More knowledge is needed about mechanisms of connection between smoking addiction and MHCs, which may be environmental or social (e.g., tobacco norms and smoking exposure among peers and in treatment facilities), result from common genetic predispositions, brain functioning or other risk factors, or stem from behavior associations or symptom management habits (e.g., alcohol use as a trigger for smoking and vice versa and symptom self-medication) [5, 43, 44]. Reasons for comorbidity between tobacco addiction and MHCs also may differ for specific disorders. Determining the best method of assessing and identifying callers with MHCs will aid in and inform research on whether tailored treatments improve outcomes for callers with MHCs and how best to tailor treatment.

Experts have put forth recommendations for tailoring treatment for people with MHCs. These include provision of more intensive counseling, higher doses of NRT or combination therapy, and cessation medications that also target mood (i.e., varenicline and bupropion) [2, 11, 15, 28, 39]. Care comanagement with mental health providers has been suggested, particularly with psychiatric medication prescribers. Since nicotine impacts how some common psychiatric medications are metabolized, medications may need adjustment during and after quitting [11, 28, 45, 46]. Treatment providers may also need to address tobacco users' concerns about weight gain, which could be complicated by medication side effects and higher rates of inactivity, focus on beliefs about self-medication with tobacco and alternative coping strategies, and use concrete smaller goals for individuals with lower cognitive functioning [2, 15, 28, 39]. Finally, changes in psychiatric symptoms should be monitored during quitting [2, 17, 28]. Strategies such as reducing to quit, pairing tobacco quitline treatment with a brief alcohol intervention, or combining quitline and community treatment may also warrant additional research [2, 31].

More research is needed to test which recommendations yield improvements in quit outcomes, particularly in quitline settings. Several quitline studies provide relevant findings to inform future research. Outcomes were similar for tobacco users with and without a psychiatric history (identified via chart review) who received 12 weeks of varenicline and phone and/or web-based behavioral treatment [8]. Given these findings, future research should examine whether varenicline is particularly effective for callers with MHCs. Second, a prospective study of callers to the Victorian Quitline in Australia provides some support for a quitline-doctor comanagement model; 83% of callers who self-disclosed doctor-diagnosed Depression believed it would be beneficial to involve their doctor in their quit attempt, and those receiving comanagement were more likely to make a quit attempt [46]. Finally, a promising randomized controlled trial in the Dutch National Quitline examined whether a mood management component integrated into standard quitline treatment improved outcomes over the standard program for callers with past major Depression; they found the additional sessions and content increased prolonged abstinence rates at 6 and 12 months but did not impact a recurrence of depressive symptoms, and differences in 7-day point prevalence abstinence rates were not significant [47].

### 4.1. Limitations

A number of limitations should be considered when interpreting these findings. First, MHCs in this study were assessed by asking callers whether they currently had one of seven MHCs. This assessment method may not have captured individuals with undiagnosed MHCs, disorders not assessed (e.g., anxiety disorders other than GAD or PTSD), subclinical symptoms, or a diagnosis they preferred not to report. Failure to capture all individuals with clinical or subclinical MHCs, or the potential for false positives from people who self-reported “yes” to an MHC but may not have been screened positive for symptoms, may have impacted results. For example, previous research suggests that individuals with subclinical levels of MHCs are more likely to smoke [48], and subclinical levels may reduce the likelihood of quit success [49]. While rates of any reported MHCs were higher in this sample (46%) compared to those estimated in the general population of smokers in the United States (30%, excluding SUD [1]), the high rate of MHCs is not surprising since many state quitlines target underserved populations, and rates of MHCs are higher in smokers [1, 6]. The assessment method used may still underidentify MHCs in our sample. For example, SUD appears likely to be underreported by participants (6.7% in this study versus 23% of callers when amount of drinking was assessed in a New York State Smokers' Quitline study [31]). When developing the questions used in this study, an expert quitline workgroup convened by NAQC considered assessment options while weighing time and other treatment considerations [2]. However, as the workgroup noted, more research is needed to identify the most effective and efficient assessment approach for the more than 400,000 annual tobacco quitline callers.

Second, many states offer a one-call program instead of or in addition to a multiple-call program. We did not have sufficient samples to examine outcomes for one-call program enrollees with and without a MHC. Based on recommendations for more intensive treatment [2, 28], we expect one-call programs may be less effective for callers with MHCs. Third, our study focused on only three states, which may limit the generalizability of findings. However, our findings concur with unpublished data presented at conferences for more than seven other states [21, 22, 29]. Fourth, the 7-month survey response rate was 41%. This is in line with state quitline evaluation survey response rates reported elsewhere [18, 32] but again may impact the generalizability of findings. All analyses included weights to adjust for survey response bias to improve the representativeness of results. Finally, our outcome measure was self-reported abstinence from tobacco for 30 or more days at the time of the 7-month survey; we did not examine prolonged abstinence and did not have access to biochemically verified quit status (i.e., cotinine or carbon monoxide data). We used this outcome metric because it is the standard for evaluating quitline outcomes in North America [34]. Furthermore, the Society for Research on Nicotine and Tobacco Subcommittee on Biochemical Verification has recommended that biochemical verification of abstinence is not necessary in large-scale studies with no face-to-face contact where data collection is done by mail, telephone, or internet [50].

## 5. Conclusion

More research is needed to address the best approach to treatment for quitline callers with MHCs, to determine what information should be assessed to provide the best care to quitline callers with MHCs (e.g., diagnoses, symptoms, and current medications), and to understand relapse profiles, reasons, and timing for this population. Given that half of quitline callers report a MHC and these callers had significantly lower quit rates, development and testing of more intensive or tailored programs to improve outcomes are warranted. Mental health and tobacco control communities should continue to develop partnerships to address this health disparity.

## Figures and Tables

**Table 1:** Quitline services for tobacco users enrolled in the Nebraska, North Carolina, or Maryland state tobacco quitlines.

Services	State quitline		
	Nebraska	North Carolina	Maryland
Registration dates included	1 August 2012–31 October 2012	Evaluation 1: 1 January 2012–30 June 2012 Evaluation 2: 8 August 2012–31 May 2013	1 December 2012–31 May 2013

7 M responders/number in sample	136/342	Evaluation 1: 827/1,966 Evaluation 2: 753/1,875	1,546/3,463

Evaluation sample selection	Census	Evaluation 1: oversampled Medicaid Evaluation 2: oversampled Medicaid, pregnant, MHCs	Census; random sampling (March–May 2013)

One-call program^a^	All tobacco users	All tobacco users	All tobacco users

Multiple-call program (Assessment and planning call plus 3-4 outbound calls)	Five-call program for tobacco users ready to quit in the next 30 days	Four-call program for tobacco users ready to quit in the next 30 days	Four-call program for tobacco users ready to quit in the next 30 days

Ten-call program	Pregnant tobacco users	Pregnant tobacco users	Pregnant tobacco users

Web Coach (Interactive online complement to phone coaching)	All phone program participants	All phone program participants	All phone program participants

Stand-alone web-based tobacco cessation Program^a^	Not offered	For tobacco users who preferred to receive only online support (starting 1 January 2012)	For tobacco users who preferred to receive only online support (starting 12 January 2012)

Direct Mail Order (DMO) nicotine replacement therapy (NRT)	(i) Not offered (ii) Proof of quitline enrollment and completion of a program call was a component for some Medicaid participants to receive NRT or medications through their pharmacy benefits manager	(i) Eight-week supply of patch, lozenge, or gum to multiple-call enrollees in the following groups: (a) all (1 January 2012–20 May 2012) (b) uninsured (starting 19 December 2012) (c) Orange County residents (starting 13 February 2013) (ii) Two-week (starting 13 February 2013) supply of patch, lozenge, or gum for multiple-call enrollees who were insured (expanded to 8-week supply on 22 May 2013) (iii) Eight-week supply of patches for multiple-call enrollees with state employees' health insurance (duration of study timeframe)	Four-week supply of patch, lozenge, or gum to all multiple-call (once every 12 months)

Note: 7 M = 7-month survey; MHCs = mental health conditions (conditions assessed: Attention Deficit Hyperactivity Disorder, Bipolar Disorder, Depression, Drug or Alcohol Use Disorder (or Substance Use Disorder), Generalized Anxiety Disorder, Posttraumatic Stress Disorder, and Schizophrenia).

^a^This study focused on callers who enrolled in a multiple-call telephone program. Individuals who selected the one-call program or the stand-alone web-based tobacco cessation program were not included. Limited 7-month evaluation data was available for these groups because a small minority selected these services and only Maryland collected follow-up data for the stand-alone web-based program during this timeframe.

**Table 2:** Baseline characteristics among multiple-call program callers with and without self-reported MHCs in three states.

Baseline data	Total (*N* = 3,132)	No MHCs (*N* = 1,697, 54.2%)	One or more MHCs (*N* = 1435, 45.8%)	*P* value^a^
	Weighted %	Weighted %	Weighted %	
Age – mean (SD)	46.3 (13.2)	47.0 (13.8)	45.5 (12.4)	0.0134
Gender				
Male	36.6	39.8	32.7	0.0005
Female	63.4	60.2	67.3	
Education				
Less than high school	21.0	18.4	24.1	0.0007
GED	6.4	5.7	7.1	
High school degree	28.7	31.4	25.6	
Some college/trade school	27.5	27.2	27.9	
College/trade school degree	16.4	17.4	15.2	
Race/ethnicity				
White, non-Hispanic	59.7	52.0	68.9	<0.0001
Black, non-Hispanic	33.2	39.9	25.1	
Hispanic	2.4	2.9	1.9	
Other, non-Hispanic	4.7	5.2	4.1	
Insurance status				
Medicaid	22.1	16.5	28.8	<0.0001
Uninsured	39.7	42.2	36.7	
Private	23.9	29.0	17.8	
Medicare	14.3	12.2	16.7	
Tobacco type^b^				
Cigarette	97.6	97.0	98.1	0.0978
Cigar	2.7	2.6	2.9	0.7038
Pipe	0.3	0.4	0.3	0.7091
Smokeless	2.0	2.2	1.7	0.4111
Other	0.8	0.5	1.2	0.1066
CPD – mean (SD)	18.6 (11.3)	17.7 (10.5)	19.8 (12.0)	<0.0001
0–10	30.5	32.8	27.9	0.0003
11–20	46.7	47.6	45.6	
21–30	12.7	11.9	13.6	
31+	10.1	7.8	12.9	
Time to first use				
<5 min	52.9	49.0	57.6	0.0003
6–30 min	28.7	30.3	26.9	
31–60 min	9.0	9.8	8.1	
60 min+	9.3	10.9	7.4	
Dependence index^c^	2.6 (0.8)	2.6 (0.8)	2.7 (0.8)	<0.0001
Below median (1–2.5)	52.3	55.9	47.9	0.0001
Above median (3-4)	47.7	44.1	52.1	
Chronic health conditions				
Any of 4 conditions	45.9	38.3	54.9	<0.0001
Asthma	18.4	11.4	26.5	<0.0001
Diabetes	14.9	12.6	17.5	0.0004
COPD	19.2	14.5	24.7	<0.0001
CAD	9.2	8.2	10.4	0.0481

Note: MHCs = mental health conditions (conditions assessed: Attention Deficit Hyperactivity Disorder, Bipolar Disorder, Depression, Drug or Alcohol Use Disorder (or Substance Use Disorder), Generalized Anxiety Disorder, Posttraumatic Stress Disorder, and Schizophrenia); GED = General Education Development; CPD = cigarettes per day; time to first use = time to first tobacco use after waking; COPD = chronic obstructive pulmonary disease; CAD = coronary artery disease.

^a^ *P* values computed using proc surveylogistic for categorical variables and proc surveyreg for continuous variables. *P* values tested for significant differences in baseline variable proportions or mean values for callers who reported no MHCs versus 1 or more MHCs; a cutoff of *P* < 0.05 was used for statistical significance. Missing data are excluded for each variable.

^b^These are not mutually exclusive categories. Participants could choose multiple tobacco products, if appropriate.

^c^Four-point scale index to represent tobacco dependence level based on cigarettes per day and time to first tobacco use after waking. Higher scores on the index represent a higher level of tobacco dependence.

**Table 3:** Program engagement and 7-month survey outcomes among multiple-call program callers with and without self-reported MHCs in three states.

Program engagement and 7-month survey responses	Total (*N* = 3,132)	No MHCs (*N* = 1,697, 54.2%)	One or more MHCs (*N* = 1435, 45.8%)	*P* value^a^
	Weighted %	Weighted %	Weighted %	
Program engagement				
Calls completed - mean (SD)	1.8 (1.0)	1.8 (1.0)	1.9 (1.1)	0.0020
1-2	80.1	82.3	77.5	0.0008
3+	19.9	17.7	22.5	
Received NRT from quitline	74.6	79.2	69.1	<0.0001

Seven-month survey responses				
Used cessation medication to help quit since enrollment	74.0	73.7	74.4	0.7196
Satisfied with quitline program	92.9	93.1	92.6	0.6376
Quit 7 days	31.9	35.5	27.6	<0.0001
Quit 30 days	26.9	31.0	22.0	<0.0001

Note: MHCs = mental health conditions (conditions assessed: Attention Deficit Hyperactivity Disorder, Bipolar Disorder, Depression, Drug or Alcohol Use Disorder (or Substance Use Disorder), Generalized Anxiety Disorder, Posttraumatic Stress Disorder, and Schizophrenia).

^a^ *P* values computed using proc surveylogistic for categorical variables and proc surveyreg for continuous variables. *P* values tested for significant differences in program engagement and 7-month survey variable proportions or mean values for callers who reported no MHCs versus 1 or more MHCs; a cutoff of *P* < 0.05 was used for statistical significance. Missing data are excluded for each variable.

**Table 4:** Multivariable models of the relationship of 30-Day tobacco abstinence and MHC status, by any conditions versus none (Model 1) and by condition group versus none (Model 2) in three states.

	Model 1		Model 2	
	Quit 30+ days^a^ (*N* = 2,870)		Quit 30+ days^a^ (*N* = 2,870)	
	AOR (95% CI)	*P* value	AOR (95% CI)	*P* value
Age	0.99 (0.99–1.00)	0.17	0.99 (0.98–1.00)	0.11
Gender				
Male	ref	0.12	ref	0.08
Female	0.85 (0.69–1.04)		0.83 (0.67–1.02)	
Education				
Less than high school	ref		ref	
GED	0.79 (0.48–1.29)		0.78 (0.48–1.27)	
High school degree	1.08 (0.81–1.42)	0.15	1.08 (0.81–1.43)	0.14
Some college/trade school	0.78 (0.58–1.04)		0.78 (0.58–1.04)	
College/trade school degree	0.91 (0.66–1.26)		0.91 (0.65–1.26)	
Race/ethnicity				
White, non-Hispanic	ref	0.69	ref	0.69
Black, non-Hispanic	0.88 (0.70–1.09)		0.88 (0.70–1.09)	
Hispanic	0.93 (0.45–1.95)		0.91 (0.44–1.91)	
All other races, non-Hispanic	0.90 (0.55–1.48)		0.91 (0.55–1.49)	
Insurance status				
Private insurance	ref	0.03	ref	0.048
Medicare-insured	0.74 (0.54–1.00)		0.75 (0.55–1.02)	
Medicaid-insured	0.68 (0.52–0.89)		0.69 (0.53–0.90)	
Uninsured	0.83 (0.64–1.08)		0.84 (0.64–1.09)	
Dependence index^b^	0.78 (0.69–0.89)	<0.001	0.78 (0.69–0.89)	<0.001
Chronic health condition				
None	ref	0.14	ref	0.12
Any of 4	1.18 (0.95–1.46)		1.19 (0.96–1.48)	
Calls completed	1.31 (1.20–1.42)	<0.001	1.31 (1.21–1.43)	<0.001
Use of cessation medications				
Reported no use	ref	0.53	ref	0.54
Used medications	0.93 (0.73–1.17)		0.93 (0.73–1.18)	
Mental health condition status (0 versus 1+)				
None reported	ref	<0.001	n/a	
One or more	0.63 (0.51–0.77)			
Mental health condition group				
None reported			ref	
Group 1: Schizophrenia/Bipolar Disorder			0.53 (0.39–0.73)	
Group 2: Depression/Anxiety/PTSD (no Group 1)	n/a		0.70 (0.55–0.90)	<0.001^c^
Group 3: ADHD or SUD (no Group 1 or 2)			0.44 (0.22–0.87)	

Note: *N* = total sample size; AOR = Adjusted Odds Ratio; CI = Confidence Interval; ref = reference group.

^a^30-day point prevalence abstinence at 7-month survey.

^b^Four-point scale index to represent tobacco dependence level based on cigarettes per day and time to first tobacco use after waking. Higher scores on the index represent a higher level of tobacco dependence.

^c^The three MHC groups did not significantly differ in likelihood of being quit (Group 1 versus 2: adjusted OR = 0.76 (95% CI = 0.54–1.06), *P* = 0.11; Group 1 versus 3: adjusted OR = 1.21 (95% CI = 0.58–2.51), *P* = 0.61; Group 2 versus 3: adjusted OR = 1.60 (95% CI = 0.79–3.22), *P* = 0.19).

Notes: models also included state as a fixed effect. Callers with missing data on one or more model variables were excluded from the model. *P* values indicate whether variables were a significant predictor of quit status in the multivariable models; a cutoff of *P* < 0.05 was used for statistical significance.
